# Supplementary material for: A set of multi-entry identification keys to African frugivorous flies (Diptera, Tephritidae)
Source: Zookeys. 2014 Jul 24;(428):97–108. doi: 10.3897/zookeys.428.7366 (PMC4143993; doi:10.3897/zookeys.428.7366)
Supplement: Supplementary material 4 — Key to Capparimyia [file zookeys-428-097-s004.zip › SF4_ZooKeys_key to Capparimyia/lucid_player/help/help/whgdata/whlstf3.htm]

Search Words List


**<<**

item **1** **2** **3** **4** **5** **6** **7**   
items **1** **2** **3** **4** **5** **6**   
**itself**   
  
  
**j2re**   
java **1** **2** **3** **4** **5**   
**join**   
jre **1** **2**   
**jump**   
jvm **1** **2**   
  
  
keep **1** **2** **3**   
keeping **1** **2**   
**kept**   
key **1** **2** **3** **4** **5** **6** **7** **8** **9** **10** **11** **12** **13** **14** **15** **16** **17** **18** **19** **20** **21** **22** **23** **24**   
**keyat**   
**keyboard**   
**keying**   
keys **1** **2** **3** **4** **5** **6** **7** **8** **9** **10** **11** **12** **13**   
**kinds**   
know **1** **2**   
**knowledge**   
**known**   
  
  
**lack**   
language **1** **2** **3**   
**languages**   
**large**   
**largest**   
latest **1** **2** **3**   
leaf **1** **2**   
learn **1** **2**   
**leave**   
**leaves**   
**leaving**   
left **1** **2**   
length **1** **2**   
lengths **1** **2**   
less **1** **2** **3**   
like **1** **2** **3**   
**likely**   
**link**   
links **1** **2**   
linux **1** **2** **3**   
list **1** **2** **3** **4** **5** **6** **7** **8** **9** **10** **11** **12** **13** **14**   
listed **1** **2** **3**   
lists **1** **2** **3** **4** **5** **6** **7**   
loads **1** **2**   
located **1** **2** **3** **4** **5**   
**location**   
**logging**   
**logically**   
**login**   
**look**   
lower **1** **2**   
lucid **1** **2** **3** **4** **5** **6** **7** **8** **9** **10** **11** **12** **13** **14** **15**   
lucid3 **1** **2** **3** **4** **5** **6** **7** **8** **9** **10** **11** **12** **13** **14** **15** **16** **17** **18** **19**   
lucidcentral **1** **2** **3** **4** **5** **6**   
  
  
mac **1** **2**   
machine **1** **2** **3** **4**   
**machine2**   
macintosh **1** **2**   
**mail**   
main **1** **2** **3** **4** **5**   
**maintains**   
make **1** **2** **3** **4** **5** **6** **7**   
**maker**   
**manage**   
**manually**   
match **1** **2** **3** **4** **5** **6** **7** **8** **9** **10**   
**matches**   
matching **1** **2** **3**   
matrix **1** **2** **3**   
mean **1** **2**   
**meaning**   
**means**   
member **1** **2**   
members **1** **2**   
menu **1** **2** **3** **4** **5** **6** **7** **8** **9** **10** **11** **12**   
**menus**   
**message**   
method **1** **2**   
**methods**   
**microsoft**   
**mineral**   
minerals **1** **2** **3**   
**minor**   
**misidentification**   
**misinterpretation**   
misinterpretations **1** **2**   
mode **1** **2** **3** **4** **5**   
modes **1** **2**   
**monographs**   
**morphological**   
**mouth**   
moved **1** **2** **3**   
**much**   
multi **1** **2**   
multimedia **1** **2**   
multistate **1** **2**   
must **1** **2** **3** **4**   
**my**   
  
  
name **1** **2**   
**names**   
**narrowed**   
**narrowing**   
**navigation**   
necessary **1** **2**   
need **1** **2** **3** **4** **5** **6** **7** **8**   
**nested**   
**network**   
**never**   
new **1** **2**   
next **1** **2** **3** **4** **5** **6**

**>>**
